# Supplementary material for: Short-Term Pain Evolution and Treatment Success of Pulpotomy as Irreversible Pulpitis Permanent Treatment: A Non-Randomized Clinical Study
Source: J Clin Med. 2022 Jan 31;11(3):787. doi: 10.3390/jcm11030787 (PMC8836521; doi:10.3390/jcm11030787)
Supplement: Supplementary file 1 [file jcm-11-00787-s001.zip › jcm-1574230-supplementary.pdf]

SUPPLEMENTARY MATERIALS

Practitioner pain scale

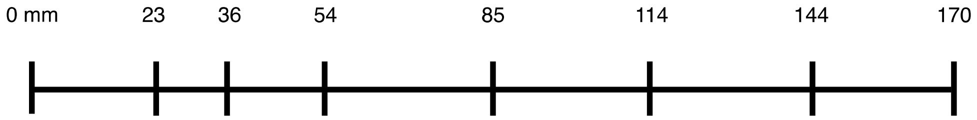

Patient pain scale

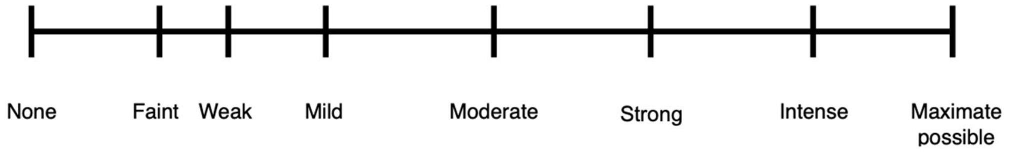

Figure S1. Practitioner and patient pain scales

Table S1. Detailed follow-up evaluation for each patient in *test* group (NA: non applicable, clinical failure < 12 months)

Patient Code	Global Follow-up (months)	Clinical Success	Initial PAI score	Final PAI score	Global treatment success
P002	56	Yes	1	1	Yes
P003	55	Yes	2	1	Yes
P008	48	Yes	1	1	Yes
P009	48	Yes	2	2	Yes
P010	47	Yes	1	2	Yes
P014	46	Yes	2	1	Yes
P005	45	Yes	1	1	Yes
P015	42	Yes	1	1	Yes
P022	37	Yes	1	1	Yes
P023	36	Yes	2	1	Yes
P001	34	Yes	1	1	Yes
P013	34	No	1	3	No
P007	29	Yes	1	1	Yes
P029	29	Yes	3	2	Yes
P034	26	Yes	1	2	Yes
P035	26	Yes	1	1	Yes
P039	24	Yes	1	2	Yes
P042	24	Yes	1	2	Yes
P041	23	Yes	1	1	Yes
P012	21	Yes	1	1	Yes

P043	20	Yes	1	4	No
P045	20	Yes	3	2	Yes
P046	20	Yes	2	2	Yes
P049	16	Yes	2	1	Yes
P050	16	Yes	2	2	Yes
P051	16	Yes	3	2	Yes
P047	14	No	1	2	No
P053	13	Yes	2	1	Yes
P057	13	Yes	2	1	Yes
P054	12	No	2	2	No
P056	12	Yes	1	1	Yes
P040	2	No	2	NA	No
P011	1	No	3	NA	No
P028	1	No	2	NA	No
P036	1	No	2	NA	No

Table S2. Detailed follow-up evaluation for each patient in *control* group (NA: non applicable, clinical failure < 12 months)

Patient Code	Global Follow-up (month)	Clinical Success	Initial PAI score	Final PAI score	Global treatment success
P101	54	Yes	1	1	Yes
P102	54	Yes	1	2	Yes
P106	51	Yes	3	4	No
P104	50	Yes	3	3	Yes
P107	49	Yes	2	3	No
P108	47	Yes	1	1	Yes
P111	31	Yes	1	2	Yes
P113	29	Yes	1	2	Yes
P115	28	No	1	3	No
P116	27	Yes	1	1	Yes
P117	26	Yes	2	3	No
P118	26	Yes	2	3	No
P125	26	Yes	2	2	Yes
P123	25	Yes	2	3	No
P120	24	Yes	1	3	No
P128	17	Yes	3	3	Yes
P129	17	Yes	1	1	Yes
P131	17	Yes	2	2	Yes

P132	17	Yes	1	1	Yes
P133	17	Yes	1	2	Yes
P134	17	Yes	3	2	Yes
P112	16	No	3	5	No
P135	16	Yes	2	2	Yes
P136	15	Yes	1	2	Yes
P137	14	Yes	2	1	Yes
P138	13	Yes	2	3	No
P141	13	Yes	4	2	Yes
P139	12	Yes	3	3	Yes
P140	12	Yes	4	3	Yes
P130	9	No	4	NA	No
